# Biosurfactant as an Enhancer of Geologic Carbon Storage: Microbial Modification of Interfacial Tension and Contact Angle in Carbon dioxide/Water/Quartz Systems

**DOI:** 10.3389/fmicb.2017.01285

**Published:** 2017-07-11

**Authors:** Taehyung Park, Hyun-Woo Joo, Gyeong-Yeong Kim, Seunghee Kim, Sukhwan Yoon, Tae-Hyuk Kwon

**Affiliations:** ^1^Department of Civil and Environmental Engineering, Korea Advanced Institute of Science and Technology Daejeon, South Korea; ^2^Department of Civil Engineering, University of Nebraska-Lincoln, Lincoln NE, United States

**Keywords:** Geologic carbon storage, *Bacillus subtilis*, surfactin, interfacial tension, contact angle, sweep efficiency

## Abstract

Injecting and storing of carbon dioxide (CO_2_) in deep geologic formations is considered as one of the promising approaches for geologic carbon storage. Microbial wettability alteration of injected CO_2_ is expected to occur naturally by microorganisms indigenous to the geologic formation or microorganisms intentionally introduced to increase CO_2_ storage capacity in the target reservoirs. The question as to the extent of microbial CO_2_ wettability alteration under reservoir conditions still warrants further investigation. This study investigated the effect of a lipopeptide biosurfactant—surfactin, on interfacial tension (IFT) reduction and contact angle alteration in CO_2_/water/quartz systems under a laboratory setup simulating *in situ* reservoir conditions. The temporal shifts in the IFT and the contact angle among CO_2_, brine, and quartz were monitored for different CO_2_ phases (3 MPa, 30°C for gaseous CO_2_; 10 MPa, 28°C for liquid CO_2_; 10 MPa, 37°C for supercritical CO_2_) upon cultivation of *Bacillus subtilis* strain ATCC6633 with induced surfactin secretion activity. Due to the secreted surfactin, the IFT between CO_2_ and brine decreased: from 49.5 to 30 mN/m, by ∼39% for gaseous CO_2_; from 28.5 to 13 mN/m, by 54% for liquid CO_2_; and from 32.5 to 18.5 mN/m, by ∼43% for supercritical CO_2_, respectively. The contact angle of a CO_2_ droplet on a quartz disk in brine increased: from 20.5° to 23.2°, by 1.16 times for gaseous CO_2_; from 18.4° to 61.8°, by 3.36 times for liquid CO_2_; and from 35.5° to 47.7°, by 1.34 times for supercritical CO_2_, respectively. With the microbially altered CO_2_ wettability, improvement in sweep efficiency of injected and displaced CO_2_ was evaluated using 2-D pore network model simulations; again the increment in sweep efficiency was the greatest in liquid CO_2_ phase due to the largest reduction in capillary factor. This result provides novel insights as to the role of naturally occurring biosurfactants in CO_2_ storage and suggests that biostimulation of biosurfactant production may be a feasible technique for enhancement of CO_2_ storage capacity.

## Introduction

Geological carbon storage (GCS) is being pursued as one of the promising solutions to stabilize the atmospheric CO_2_ concentration (White et al., 2003; Solomon et al., 2009). CO_2_ storage in geologic formations is achieved by injecting CO_2_ into depleted oil and gas reservoirs or deep saline aquifers (Stevens et al., 2001; Bachu and Adams, 2003); the injected CO_2_ is expected to be immobilized by several mechanisms including capillary trapping, solubility trapping, and mineral trapping (Suekane et al., 2008; Shogenova et al., 2009; Juanes et al., 2010). Evaluating the stability of capillary trapping and the corresponding CO_2_ storage capacity in geologic formations is a challenging task. For this purpose, the interfacial tension (IFT) between CO_2_ and water/brine and the wettability of CO_2_ to different minerals are the pre-requisites to answer relevant questions; for instance, how much brine will be swept by the CO_2_ invasion in brine-saturated porous networks and how far CO_2_ will propagate away from an injection well (Bachu et al., 1994; Juanes et al., 2010). Therefore, CO_2_-brine IFT and CO_2_-brine-mineral contact angle under varying temperature and pressure conditions are critically important for the accurate prediction of sweep efficiency, fluid injectivity, and storage capacity, which contributes to the success of GCS operation (Chiquet et al., 2007; Bachu and Bennion, 2008; Georgiadis et al., 2010; Cao et al., 2016).

Various methods, including the injections of acid gas, carbonated water, and surfactant into target formations, have been studied to increase the CO_2_ storage capacity and injectivity (Shah et al., 2008; Sohrabi et al., 2012; Kim and Santamarina, 2014). In particular, use of synthetic surfactants has been proposed for the enhanced GCS by reducing the capillary pressure of a CO_2_-water-rock system and increasing the pore-scale sweep efficiency of CO_2_ (Kim and Santamarina, 2014). In the meantime, biosurfactants have been drawing more attention as an alternative to the synthetic surfactants for their biodegradability, biocompatibility, ecological suitability, high specificity, low toxicity, and production on the basis of renewable resources (Desai and Banat, 1997; Rosenberg and Ron, 1999; Joshi et al., 2008). For example, rhamnolipids produced from *Pseudomonas aeruginosa* were reported to be four times more effective in hexadecane removal, and glycolipids from *Rhodococcus* species 413A were 50% less toxic than a synthetic surfactant, Tween 80, in naphthalene solubilization tests (Brusseau et al., 1995; Kanga et al., 1997). Reportedly, natural occurrence of biosurfactants by biosurfactant-producing bacteria is fairly common in subsurface environments, such as oil and gas reservoirs and even natural gas hydrate deposits (Banat, 1995; Lanoil et al., 2001). As microbial activities in some GCS candidate sites have been confirmed (Morozova et al., 2010; Lavalleur and Colwell, 2013), a recent study by Peet et al. (2015) suggested the possibility of microbial growth at GCS sites and that microbial activity would potentially alter the fate and behavior of injected CO_2_ in the deep subsurface. The authors also confirmed the growth of surfactant-producing *Bacillus* strains under supercritical CO_2_ (Peet et al., 2015). Moreover, most of the pilot-scale GCS projects are currently carried out under high pressure environments with supercritical CO_2_ phase (e.g., 59°C and 15 MPa in Frio Brine Pilot Project, United States; 90°C and 18 MPa in In Salah CO_2_ Storage Project, Algeria; 85°C and 10 MPa in CO2CRC Otway Project, Australia; 60°C and 15 MPa in Weyburn-Midale Carbon Dioxide Project, Canada; Riding and Rochelle, 2005; Doughty et al., 2008; Boreham et al., 2011; Preisig and Prévost, 2011). However, the lack of experimental data on the variations of interfacial properties by production of biosurfactants under such high-pressure conditions hinders us from developing a predictive model of CO_2_-brine flow dynamics in porous media.

We herein investigated the roles of biosurfactants for altering CO_2_-brine-rock interfacial properties as an enhancer of GCS, by quantifying the extent of CO_2_-brine IFT reduction and CO_2_ wettability modification for different CO_2_ phases at high pressure conditions. The model microorganism *B. subtilis* was cultured and stimulated to produce biosurfactant (surfactin) under high pressure conditions. At the same time, the alteration of the CO_2_-brine IFT and the contact angle of CO_2_-brine-quartz for different phases of CO_2_, i.e., gas, liquid, and supercritical phases was monitored over the courses of the microbial growth and biosurfactant production. We then examined the effect of altered interfacial properties on the CO_2_ sweep efficiency in porous media by conducting pore network simulations, which was followed by discussions on the use of *in situ* biosurfactant production to improve the GCS operation.

## Materials and Methods

### Model Microorganism

In this study, *B. subtilis* strain ATCC6633 was selected as the model organism for investigation of biosurfactant production under high pressure conditions. *Bacillus* spp. are among the most commonly found in soil and aquatic environments and *B. subtilis* endospores have been found in even the most unlikely environments to harbor life, such as rocks, dusts and aquatic environments (Nicholson, 2002). *Bacillus* spp. are facultative anaerobic bacteria well-known for their survivability under extreme pH, salinity or high pressure and high temperature conditions (Zobell and Johnson, 1948; Yakimov et al., 1995; Wilks et al., 2009; Willenbacher et al., 2015). Further, *Bacillus* spp. have been found in oil reservoirs in Japan (i.e., Minami-aga (Niigata) oil field, which is 2150 m deep and 106°C; Yabase (Akita) oil field, which is 1700 m and 95°C; Kato et al., 2001) and oil reservoirs in Atlantic Ocean, offshore Rio de Janeiro, Brazil, which is approximately 2650 m deep and 60°C (Cunha et al., 2006). GCS projects often target deep subsurface geologic formations aforementioned, where anoxic or microoxic conditions are prevalent, high temperature and acidic pH are often observed, and CO_2_ mostly exists as a supercritical phase. Although supercritical CO_2_ is generally regarded as a sterilizing agent due to its membrane destabilization and cytoplasmic acidification effects, Peet et al. (2015) found the survivability of *Bacillus* spp. under supercritical CO_2_ headspace pressures of >7.3 MPa, possibly owing to the feature of spore-forming *Bacillus* spp. Therefore, use of a *Bacillus* strain as the model organism was appropriate for simulation of microbial activities at GCS sites.

### Culture Conditions and Inoculum Preparation

*Bacillus subtilis* strain ATCC6633 (KCTC2189) was acquired from the Korean Collection for Type Culture, and after resuscitation in 250 ml nutrient broth (Difco, BD, Franklin Lakes, NJ, United States), was stored as glycerol stocks (50% v/v) in –80°C freezer. Before each experiment, a freezer stock culture was resuscitated and maintained for short term in nutrient broth at 37°C.

A mineral salts medium was previously defined for enhancement of surfactin production under anoxic culture conditions (**Table 1**; Cooper and Goldenberg, 1987; Joshi and Desai, 2013). After filter-sterilization with a 0.2 μm sterile syringe filter (Chmlab Group, Terrassa-Barcelona, Barcelona, Spain), 160 mL of the fresh mineral salts medium was dispensed to each 250-mL serum bottle, which was subsequently sealed with a butyl-rubber stopper (Geo-Microbial Technologies, Inc., Ochelata, OK, United States) and an aluminum crimp. Air in the headspace was replaced with N_2_ by flushing each serum bottle with a pressurized stream of N_2_ for 40 min, followed by five cycles of N_2_ purging and subsequent vacuuming.

**Table 1:** Composition of mineral salt medium used for surfactin production.

Compound	Concentration
Glucose	40 g/L
CaCl_2_	7.0 × 10^-6^ M
Na_2_EDTA	4.0 × 10^-6^ M
MnSO_4_	1.0 × 10^-6^ M
NH_4_Cl	0.1 M
NaNO_3_	0.118 M
KH_2_PO_4_	0.03 M
Na_2_HPO_4_	0.04 M
MgSO_4_	8.0 × 10^-4^ M
FeSO_4_	4.0 × 10^-6^ M


*Deionized water (DIW) was used for the mineral salt medium preparation.*

From the starter culture in the nutrient broth, 2.5 mL was first transferred to 250 mL of fresh nutrient broth. After 20–24 h of incubation at 37°C with shaking at 200 rpm, 4 mL of this freshly grown aerobic culture was transferred to 160 mL of the fresh mineral salts medium prepared in the anoxic culture bottles. Then, this anaerobic culture was incubated for 24 h at 37°C with no shaking, and 4 mL of this anoxic culture was used to inoculate the view cell and incubated for more than 40 h and sometimes up to 180 h for surfactin production. During the course of bacterial growth and surfactin production, IFT of CO_2_-mineral salt medium and contact angle of CO_2_-mineral salt medium-quartz were measured in the view cell. In this study, the mineral salt medium (growth medium) of which the salinity was ∼1.6–1.7% was used as brine.

### Biosurfactant Extraction and FT-IR Characterization

The acid precipitation method was used to extract the biosurfactant produced by *B. subtilis* (Mukherjee et al., 2009), and the extracted biosurfactant was qualitatively characterized using Fourier transform-infrared spectroscopy (FT-IR) analysis for identifying for its functional groups and confirming the class of biosurfactant produced (Cooper et al., 1981). Although the acid precipitation method is time-consuming for its evaporative drying process, it is one of the well-known and reliable methods for surfactin extraction (Mukherjee et al., 2009). After 72 h of anaerobic fermentation of *B. subtilis*, the cell-free broth was obtained by centrifuging the anaerobic culture at 11292 × *g* for 20 min. Then, 6 N HCl was added to the cell-free supernatant until it reached to pH 2.0 and left overnight at 4°C. Afterward, the crude biosurfactant pellets were collected by centrifuging at 11292 × *g* for 20 min. These biosurfactant pellets were dissolved in DIW, extracted with chloroform:methanol (65:15), and then lyophilized to extract the biosurfactant. We extracted 6.23 mg of the biosurfactant from 100 mL of anaerobic culture. The functional groups of this extracted biosurfactant were compared with those of the standard surfactin sample (Sigma-Aldrich, St. Louis, MO, United States) via FT-IR. Our biosurfactant produced by *B. subtilis* and the reference surfactin sample were dissolved in ethanol and analyzed for the range of 400–4000 cm^-1^.

### Experimental Setup

**FIGURE 1:**
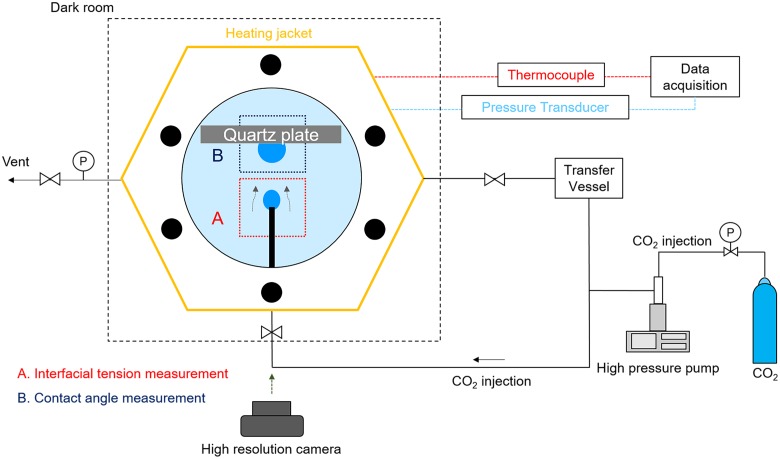
Experimental setup for measuring **(A)** interfacial tension (IFT) and **(B)** contact angle.

The experimental setup was designed to enable the measurement of interfacial properties (IFT and contact angle) under high pressure and temperature (P/T) conditions close to currently considered candidate GCS site environments, while culturing microorganisms. The measurement of IFT and contact angle was conducted in a view cell, fabricated of 304 stainless steel with an internal volume of 40 cm^3^ and equipped with two transparent quartz crystal windows, as shown in **Figure 1**. The view cell was equipped with (a) a capillary tube to generate a CO_2_ pendant drop in brine for measuring CO_2_-brine IFT (i.e., pendant drop method; see panel A in **Figure 1**) and (b) a quartz disk so as to generate CO_2_ sessile drop on that quartz disk for measuring CO_2_-brine-quartz contact angle (i.e., sessile drop method; see panel B in **Figure 1**). To improve the accuracy of the pendant drop method, the capillarity tube with the outer diameter of 1.59 mm was chosen, such that the interfacial and buoyance forces should be comparable to each other; thereby, the bond numbers (B_o_), i.e., defined as the dimensionless ratio of a buoyance force to a surface force, were controlled to be in a proper range of 0.19 to 0.83. Therefore, our measurement setup satisfies the condition for consistent and reliable IFT measurement using the pendant drop method, in which the bond number less than 1 is suggested (Berry et al., 2015).

Upon the generation of CO_2_ droplets in brine, the time-lapsed images of these droplets were acquired using a high-resolution digital single-lens reflex (DSLR) camera (Canon EOS 100d with Canon 100 mm 2.8f macro lens). For this study, compressed CO_2_ gas (a commercial 99.9% grade; Sam-O Gas Co., Daejeon, Korea) was used. A high-pressure syringe pump (500HP, Teledyne Isco, Lincoln, NE, United States) was used to pressurize CO_2_ to the target pressure and to feed and produce the CO_2_ droplets in the view cell. The transfer vessel contained and fed the fresh mineral salt medium (or brine) to the view cell, controlling the brine pressure inside the view cell. The temperature inside the view cell was controlled with a coil-type electric heater. The view cell was also instrumented with a K-type thermocouple (Hankook Electric Heater, South Korea) and a pressure transducer (PX302, Omega, Norwalk, CT, United States); thus, the temperature and pressure conditions of the fluids in high pressure cell were measured logged at the interval of 10 s using the data logger (34970A, Keysight, Santa Rosa, CA, United States).

### Experimental Procedures and Data Reduction

We evaluated the variations of CO_2_-brine IFT and CO_2_-brine-quartz contact angle at three different P/T conditions: 37°C and 3 MPa for gaseous CO_2_ phase, 28°C and 10 MPa for liquid CO_2_ phase, and 37°C and 10 MPa for supercritical CO_2_ phase, respectively. These conditions were designed to examine the effect of the phase of CO_2_, as the CO_2_ phase may vary depending on the P/T conditions of potential GCS candidate sites, including the shallow permafrost regions (e.g., Alaska north slope: ∼7 MPa, 5°C), warm coalbed reservoirs (e.g., Alabama Black Warrior Basin: ∼7 MPa, 23°C), and deep high temperature oil reservoirs (e.g., Weyburn oil field: ∼14 MPa, 50°C; Espinoza and Santamarina, 2010).

All wetting parts including tubes, fittings and the transfer vessel were autoclaved at 120°C, and the view cell was sterilized by ethyl alcohol for more than 6h before use. Initially, the *B. subtilis* inoculum of 4 mL (i.e., 10% v/v of vessel volume) was inoculated to the view cell, and then, the rest of the inner volume of the view cell was filled with the fresh mineral salts medium injected from the transfer vessel until the target pressure condition was achieved. During the fresh mineral salts medium injection, air inside was evacuated through the top fluid port that was open, to achieve no head space in the view cell. As the fluid pressure inside was then elevated to more than 3 MPa and up to 10 MPa, we confirmed that there was no head space left in the view cell. If any, the head space would be minimal because most of air was dissolved. Thereby, it was presumed to be not a strict anoxic condition but a microoxic condition. Thereafter, the overall system was kept for several minutes until the inside P/T condition was stabilized prior to creating CO_2_ droplets. Note that no mixing was provided over the course of experiments. In this study, the salinity of our mineral salt medium was approximately 1.6–1.7%; this growth medium was used as brine.

Following the pendant drop method for the IFT measurement, a pendant droplet of CO_2_ was generated at the tip of the capillary tube under the stable P/T condition (see **Figure 1A**). Upon the creation of a CO_2_ pendant drop in the mineral salt medium (or brine), the time-lapsed imageries of the drop were acquired using the digital camera at a 5-minute interval over the course of bacterial growth and surfactin production. The acquired images of a CO_2_ drop was analyzed by the axisymmetric drop-shape analysis (ADSA) method (Cheng et al., 1990; Berry et al., 2015). More details of ADSA method can be found in Berry et al. (2015). To maintain the consistency in IFT estimation, we chose the CO_2_ droplets which had been stabilized for more than 10 min after creation and of which the curvature radius at the drop apex was in the range of 0.5–0.8 mm. We noted that the droplet should be in static conditions for reliable estimation and that too big or too small droplets could often lead to some error in IFT calculations, as corroborated by previous researches (Cheng et al., 1990; Chalbaud et al., 2009).

Following the sessile drop method for the contact angle measurement, the CO_2_ droplet was created from the capillary tube under the stable P/T condition; while floating by buoyancy force, the bubble was retained by the quartz disk, so as to be in contact with the substrate that was fully submerged in brine (see **Figure 1B**). This condition represents the quartz mineral under a water-wet condition (Jung and Wan, 2012). Likewise, upon the creation of a CO_2_ sessile droplet in contact with the quartz substrate, the time-lapsed imageries of the droplet were acquired using the digital camera at a 5-minute interval over the course of bacterial growth and surfactin production. The droplet edges and profiles in the acquired images were determined by using ImageJ software, and hence the contact angles of CO_2_-brine-quartz were estimated, following the drop-snake method (Stalder et al., 2006). Herein, to maintain the consistency in contact angle estimation, the droplet images to be analyzed were chosen with the following criteria: (a) CO_2_ droplets need to be stabilized for more than 10 min after creation, (b) the droplets are required to be smaller than 5 mm in maximum circumscribed diameter, and (c) the difference in the contact angle values obtained for left and right edges is required to be less than 2%. We found that these criteria were well suited for our testing conditions, which is thought to be consistent with previous study (Jafari and Jung, 2016).

In each test, the bacterial growth of *B. subtilis* was confirmed by the turbidity of the liquid culture collected from the view cell after the completion of IFT and contact angle measurement. The production of biosurfactant was inferred from the changes in IFT and contact angle values during the experiments.

### Pore Network Model (PNM) Simulations

We performed flow simulations on two-dimensional (2D) pore network models (PNM) to further evaluate the improvement in CO_2_ displacement patterns and sweep efficiency. Herein, we compared the results with and without biosurfactant, by modifying the capillary factor. The PNM simulation was developed for the case where immiscible and non-wetting CO_2_ is injected into a porous medium saturated with a wetting fluid, herein brine that can have biosurfactant or not (Kim and Santamarina, 2014). Thereby, one case with an abiotic condition where no microbial activity without bacterial cells and surfactin was assumed, was simulated. The other extreme case was simulated for the condition where bacterial cells and surfactin was produced by microbial activities, and in this case, the interfacial properties (IFT and contact angle) that were obtained from our tests were used as input parameters. More details of this PNM simulation are described in Kim and Santamarina (2014).

The 2D 100 × 100 PNMs that consists of tubes and nodes were generated for flow simulations. All tubes had the identical length of 50 μm. The tube diameters were log-normally distributed with the mean value (d_o_) of 10 μm and the coefficient of variation (COV) of 0.6. The capillary factors were obtained from our test results for three CO_2_ phases. For instance, the capillary factor of liquid CO_2_-brine-quartz decreased from 26.6 mN/m to 6.4 mN/m, by 76.5% reduction, due to the biosurfactant production. For each case, twenty pore networks were randomly generated, and flow simulations were carried out on the generated networks while applying the identical pressure difference between the inlet and outlet (ΔP = 300 kPa) for all of the pore network simulations.

## Results

### Identification of Biosurfactant Via FT-IR Analysis

**FIGURE 2:**
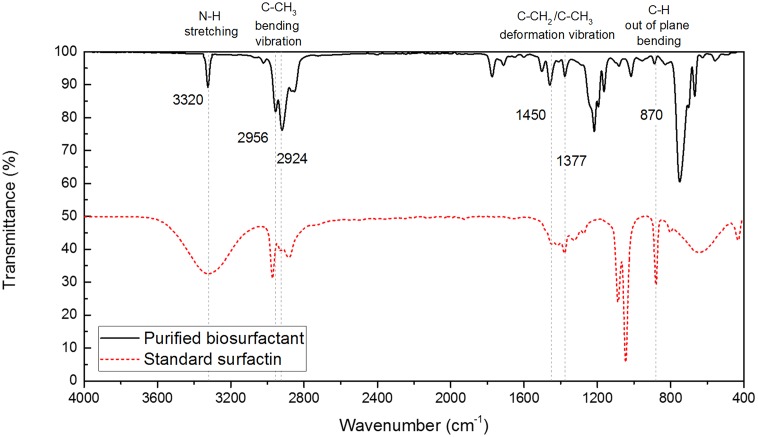
FT-IR spectra of extracted biosurfactant produced from *B. subtilis* and standard surfactin.

To compare the functional groups of the extracted biosurfactant with those of the standard surfactin sample, FT-IR analysis was conducted. **Figure 2** shows the FT-IR spectra that were recorded using a Bruker Alpha-P FT-IR spectrophotometer with the attenuated total reflectance (ATR) module. The IR spectra exhibited the N–H stretching mode at 3320 cm^-1^ (i.e., band characteristics of peptides), C–CH_3_ bending vibration mode at 2956–2924 cm^-1^ and C–CH_2_ or C–CH_3_ deformation vibration at 1456 cm^-1^ and 1377 cm^-1^. The peak corresponds to the C–H bending vibration are common in compounds with alkyl chains (Das et al., 2008; Al-Wahaibi et al., 2014). Peak found at 870 cm^-1^ was due to out of plane C–H bending, which is the characteristics of aromatic compounds (Das et al., 2008). The similarity between the two FT-IR spectra confirmed that the biosurfactant produced by *B. subtilis* with the mineral salt medium had the similar structures and functional groups to surfactin, which is also corroborated by previous studies by Joshi et al. (2008) and Willenbacher et al. (2015).

### Variations in CO_2_-Brine IFT

**FIGURE 3:**
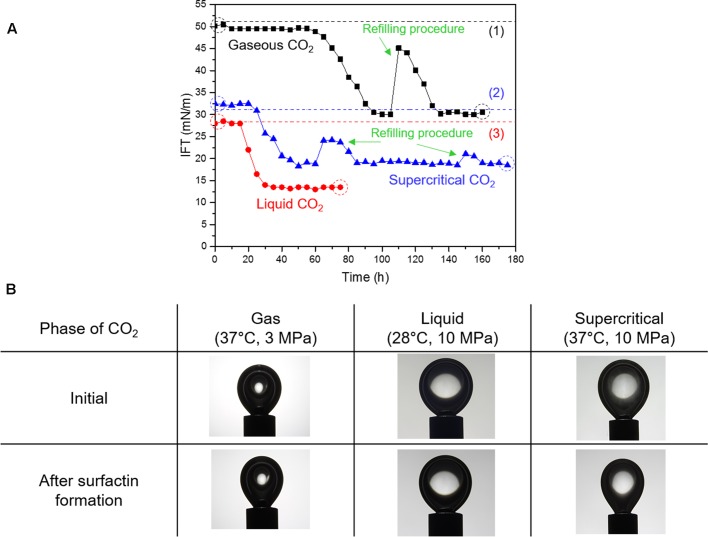
**(A)** Variations in interfacial tension (IFT) of CO_2_-brine with the surfactin production, and **(B)** the digital images of pendant CO_2_ droplets before and after the surfactin production. Note: The dotted lines indicate the IFT values from previous literature; IFT values for gaseous and supercritical CO_2_ (1,2) are from Georgiadis et al. (2010) and the IFT value for liquid CO_2_ (3) is from Hebach et al. (2002). The droplet images in panel **B** correspond to the dotted circles in panel **A**.

**Figure 3** shows the variations of the CO_2_-brine IFT during the growth of biosurfactant-forming microbe, *B. subtilis* and the images of CO_2_ pendant drops before and after surfactin production. The initial values of CO_2_-brine IFT, which were measured before any biosurfactant was produced, were consistent with the previously published data on IFT of CO_2_ and pure water (e.g., Hebach et al., 2002; Georgiadis et al., 2010), as denoted as dotted lines in **Figure 3A**. The reduction in the IFT values were observed, which indicates that the model microbes grew and the biosurfactant (or surfactin) was produced within 65, 25, and 20 h for gaseous, liquid, and supercritical CO_2_, respectively. Because of surfactin production during *B. subtilis* growth, the IFT of gaseous CO_2_ and brine decreased from 49.5 mN/m to a stable value of 30 mN/m. For liquid CO_2_, the IFT was reduced from 28.5 mN/m to 13 mN/m, then the IFT reached a plateau. For supercritical CO_2_, the IFT of supercritical CO_2_ and brine decreased from 32.5 mN/m to 18.5 mN/m.

In all cases, it appeared that the IFT values were converged to some lower limits after the rapid decrease in IFT. This implies that the concentration of surfactin reached the critical micelle concentration (CMC) or the concentration level sufficient enough to cover the surfaces of the bacterial cells. Refilling of nutrient was conducted for the cases of gaseous CO_2_ and supercritical CO_2_, where the fresh mineral salt medium of 25 mL was injected to the cell, in order to test the hypothesis that the IFT reduction may be restricted by insufficient nutrient in the brine. After the refilling procedures, the IFT values instantly increased because of the partial replacement of surfactin-containing brine with the injected fresh media. Furthermore, bacterial cells were also partly removed along with surfactin. Thereafter, the IFT values decreased owing to the surfactin production, but soon reached the same lower limits though it took some time for bacterial cells to reach sufficient amounts before producing the same level of surfactin. This indicates that the extent of the CO_2_-brine IFT reduction is limited by the CMC concentration, and its lower limits differ with CO_2_ phases. We acknowledge that the refilling process would have introduced some oxygen to the view cell, however, it was observed that the reduced IFT values after the refilling were consistent with the previous lower limits. It is presumed that small but possible oxygen feed associated with the refilling process had no or minimal impact on the results.

### Variations in CO_2_-Brine-Quartz Contact Angle

**FIGURE 4:**
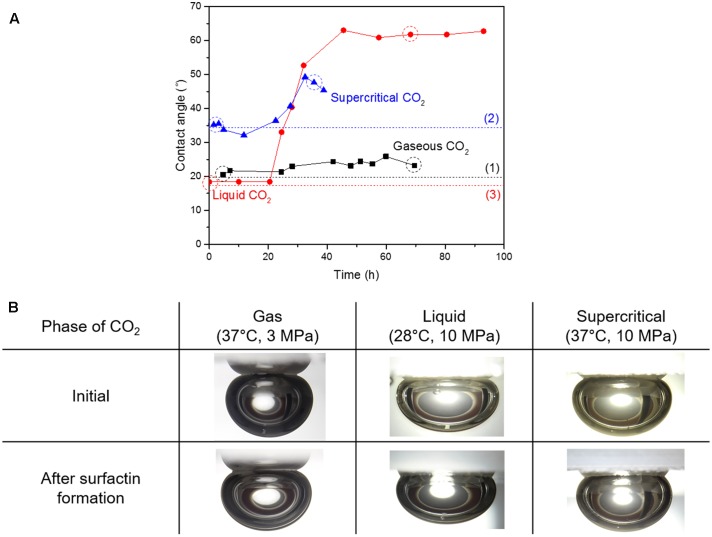
**(A)** Variations in contact angle of CO_2_-brine-quartz with the surfactin production, and **(B)** the digital images of sessile drops of CO_2_ in contact to the quartz disk before and after the surfactin production. Note: The dotted lines indicate the contact angle values from previous literature; the value for gaseous CO_2_ (1) is from Kim and Santamarina (2014) and the values for liquid and supercritical CO_2_ (2, 3) are from Sarmadivaleh et al. (2015). The droplet images in **B** correspond to the dotted circles in **A**.

**Figure 4** shows the variations of the CO_2_-brine-quartz contact angle during the growth of *B. subtilis* and the images of CO_2_ sessile drops before and after surfactin production. The initial values of CO_2_-brine-quartz contact angle, which were measured before any biosurfactant was produced, were consistent with the previously published data on the CO_2_-pure water-quartz contact angle (e.g., Kim and Santamarina, 2014; Sarmadivaleh et al., 2015), as denoted as dotted lines in **Figure 4A**. In all phases, the increases in the contact angle values were observed as the surfactin was produced with time though their magnitudes differed. The contact angle of gaseous CO_2_-brine-quartz slightly increased from 20.5° to 23.2°, by 2.7°. For liquid CO_2_, the contact angle increased from 18.4° to 61.8°, by 43.4°. For supercritical CO_2_, the contact angle increased from 35.5° to 47.7°, by 12.2°. The increments in the contact angle values as the model microbes grew and the production of biosurfactant (or surfactin) started in 28.2, 24.5, and 22.5 h for gaseous, liquid, and supercritical CO_2_, respectively. The increment of contact angle was the greatest in liquid CO_2_ but a minimal increase in the contact angle was observed in gaseous CO_2_. In the same manner with the IFT results, the contact angle values were observed to converge to some upper limits, which indicated the concentration of surfactin in brine reached the CMC or the concentration sufficient enough to cover the surface of the cells.

## Analysis and Discussion

### Effect of Surfactin Production on Interfacial Properties of CO_2_-Brine-Quartz System

The IFT of CO_2_-brine and the contact angle of CO_2_-brine-quartz were altered mainly because of surfactin produced by *B. subtilis*, ATCC6633. The IFT reduction of CO_2_-brine by *in situ* production of surfactin differed as the phase of CO_2_ varied. The greatest reduction occurred in the gaseous phase, by ∼19.5 mN/m, and the reductions in the liquid and supercritical phases were found to be similar to each other (i.e., by ∼15.5 mN/m and 14 mN/m, respectively). Meanwhile, the relative reduction ratio of IFT (i.e., defined as Δγ/γ_o_) by surfactin production were approximately 39, 54, and 43% for gaseous, liquid and supercritical CO_2_, respectively. Because the IFT of CO_2_-fluid decreases with a decrease in the density difference between the fluid and CO_2_, the liquid CO_2_, which has the highest density among three phases, has the lowest initial IFT value, and the gaseous CO_2_, which is the least dense, has the highest initial IFT value (Hebach et al., 2002; Bachu and Bennion, 2008; Chalbaud et al., 2009). In spite of the lowest initial IFT between liquid CO_2_ and brine, it is worth remarking that relative reduction in IFT between liquid CO_2_ and brine was found the greatest. Furthermore, it appeared that such relative reduction ratio in IFT caused by *in situ* surfactin production became greater as the CO_2_ density increased. This may be attributed to the higher population of surfactin molecules at the denser CO_2_-brine interface, possibly owing to the molecular interactions among CO_2_, water, and surfactin. In this study, we varied P/T conditions of experiments to investigate the wettability changes in different phase of CO_2_. However, not only the phase difference of CO_2_, but also the P/T condition can affect the microbial activity and the surfactin production and its efficiency, the exact mechanism still warrants further investigation.

Compared to our biosurfactant (or surfactin), the synthetic surfactant (surfonic POA-25R2) showed the higher efficiency in modification of CO_2_-brine IFT (Kim and Santamarina, 2014). Although the P/T conditions are different from this study, the relative reduction ratio of IFT by synthetic surfactant was approximately 34–92%. This synthetic surfactants (surfonic POA-25R2) has a branch of polyoxypropylene that is specifically designed and synthesized to enhance CO_2_ affinity, showing particularly high CO_2_-phillicity (Kim and Santamarina, 2014). While the synthetic surfactant used in their study was non-ionic, the surfactin used in this study is anionic, which could also be one of the reasons for the observed difference in surfactant activity, for instance, the greater enhancement in CO_2_ dissolution by non-ionic surfactant than by anionic surfactant (McClain et al., 1996). Meanwhile, most of biologically produced surfactants, including surfactin used in this study, have the hydrophobic parts composed of fatty acids, but their hydrophilic parts can be composed of different groups, such as amino acids, peptides, carbohydrate, carboxylic, alcohol, phosphate and/or polysaccharides (Banat, 1995; Mulligan, 2005). The other naturally occurring biosurfactants may exhibit the different efficiency in IFT reduction, owing to the structural difference in the hydrophilic parts.

The increase in the contact angle of CO_2_-brine-quartz by *in situ* production of surfactin also differed as the phase of CO_2_ varied. The greatest increase occurred in the liquid phase, by ∼43.4, and the increases by 12.2° and 2.7° were observed for the supercritical and gaseous phases, respectively. Meanwhile, the contact angle values relatively increased by the surfactin production approximately 1.16, 3.36, and 1.34 times for gaseous, liquid and supercritical CO_2_, respectively (i.e., relative increment ratio of contact angle, defined as θ_final_/θ_initial_). Despite the lowest initial contact angle for liquid CO_2_, it is worth noting that relative increment ratio of contact angle was found the greatest for liquid CO_2_ among the three phases. In addition, it was observed that such relative increment in contact angle due to the *in situ* surfactin production increased as the CO_2_ density increased. This observation is consistent with the aforementioned trend of the IFT reduction. Similar to the case of IFT, this is thought to be attributed to the higher population of surfactin molecules attached to the CO_2_-brine interface.

Compared to our biosurfactant (or surfactin), there are only few data available for synthesized synthetic surfactant (surfonic POA-25R2) applied to the contact angle among gaseous CO_2_-brine-quartz and the contact angle among liquid CO_2_-brine-quartz (Kim and Santamarina, 2014). For the similar pressure conditions, the relative increment ratio of contact angle by synthetic surfactant was approximately 1.15 for gaseous CO_2_ and 3.5 for liquid CO_2_. It was found that the efficiency of surfactin in increasing contact angle was fairly close that of the synthetic surfactant (surfonic POA-25R2).

The invasion behavior of CO_2_ into brine-saturated porous media is heavily affected by the capillary factor, i.e., defined as γ_CO2_ _-brine_ ⋅ cos θ, where γ_CO2-brine_ is the IFT between CO_2_ and brine and θ is the contact angle of CO_2_-brine-quartz. The overall reduction of capillary factors in CO_2_-brine-quartz system caused by the surfactin production was 19.5, 20.2, 14.4 mN/m in gaseous, liquid and supercritical CO_2_ phase, respectively. Meanwhile, the relative reduction ratio of capillary factor was 41.5, 76.0, and 54.4% in gaseous, liquid and supercritical CO_2_ phase, respectively. The impact of surfactin production on wettability alteration was the greatest in liquid CO_2_ and followed by supercritical and gaseous CO_2_.

Although the reported changes in the IFT and contact angle of CO_2_-brine-quartz systems are mainly attributed to the surfactin produced by *B. subtilis*, it is worth noting that bacterial cells also grow and they can interfere with CO_2_, possibly affecting the IFT and contact angle values through cell lysis, surface properties of the cells, or variation in cell viability with time. Moreover, the P/T condition can affect the microbial activity and biosurfactant production efficiency. Therefore, the comparison with the abiotic experiments with the extracted surfactin without cells are required to quantify the sole impact of surfactin on wettability alteration.

### Improvement in Sweep Efficiency of CO_2_ by Biosurfactant – PNM Simulation

**FIGURE 5:**
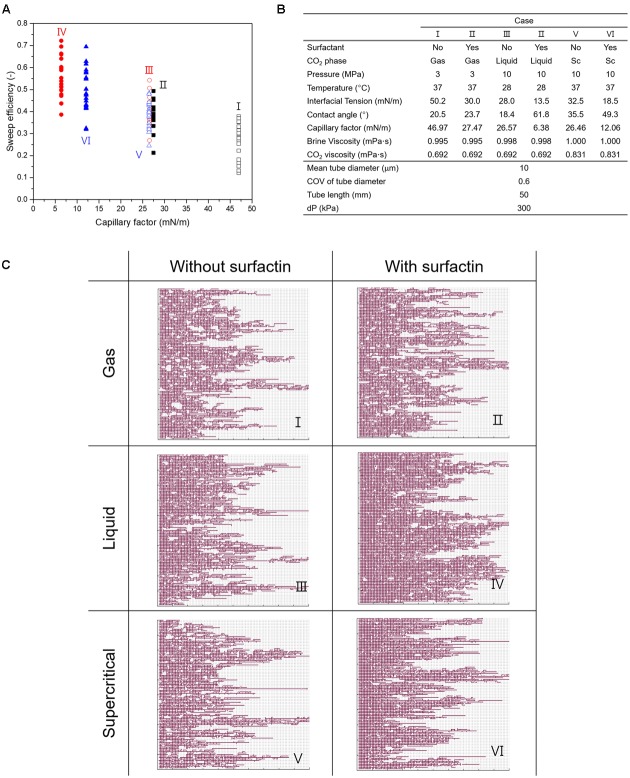
The results of PNM simulations. **(A)** Variations in sweep efficiency for the different cases. **(B)** Input parameters for PNM simulations. **(C)** The invasion patterns of CO_2_ in pore networks with different capillary factors affected by surfactin production. Cases I, III, and V represent the conditions without surfactin production, and Cases II, IV, and VI represents the conditions with surfactin production. Gray lines indicate the brine-saturated tubes in the pore networks and the red color shows the spatial distribution of CO_2_. It is noted that more CO_2_ occupies each pore network when surfactin produced.

We ran the PNM simulations for six different cases, and the input parameters are listed in **Figure 5**. As the microbes produce biosurfactant thus the capillary factor decreases, it can be seen that the sweep efficiency by CO_2_ is improved, as shown in **Figure 5**. The sweep efficiency is defined as the ratio of the volume occupied by CO_2_ to the volume of the pore network. Owing to the biosurfactant production, the average sweep efficiencies for all CO_2_ phases are improved: from 26.6 to 38.9% for gaseous CO_2_, from 38.9 to 56.6% for liquid CO_2_, and from 38.7 to 49.6% for supercritical CO_2_, respectively. The improvement is the most for liquid CO_2_ as ∼18%, and the least in supercritical CO_2_ as ∼11%. It is observed that the reduction in the capillary factor facilitates CO_2_ invasion into small pores, leading to the more even CO_2_ displacement and the higher sweep efficiency (**Figure 5B**). Therefore, as an increase in sweep efficiency leads to a decrease in the residual water saturation, more CO_2_ can be stored in a unit volume of a porous medium. Although several previous studies have shown the improvement in CO_2_ injection by use of synthetic surfactants (Eastoe et al., 2006; Kim and Santamarina, 2014), we report the modification of IFT and wettability in CO_2_-brine-mineral system by *in situ* biosurfactant production under reservoir conditions and the improvement in sweep efficiency of CO_2_ injection, for the first time to our best knowledge.

### Engineered Injection of CO_2_ and Implications to GCS

#### Engineered Injection

The sweep efficiency of CO_2_ injection can be enhanced either by increasing the viscosity of CO_2_ and/or lowering the capillary factor of CO_2_ (i.e., viscosity control vs. mobility control). Increasing the CO_2_ viscosity may help transition of the flow pattern from a viscous fingering-dominant to a more stable displacement, but can cause faster pore-pressure buildup. In contrast, lowering the capillary factor, γ_CO2-brine_ ⋅ cos θ, not only can help transition the flow pattern (from a capillary fingering to the stable displacement), but also can alleviate the pressure buildup. The rationale is as follows: the reduced capillary factor results in the smaller residual saturation of brine (because of the improved sweep efficiency), which leads to the increase in the relative permeability of CO_2_ phase. Higher relative permeability is linked to the better injectivity of CO_2_, which in turn means that less pore-pressure buildup would be resulted at a given targeted flow rate. This logic is well supported by analytical models that predict pressure buildup during the CO_2_ injection (Mathias et al., 2009; Hosseini et al., 2012). In this regard, controlling the capillary factor also has a potential to mitigate a risk of induced seismicity, which is caused from complex hydro-thermo-chemo-mechanically coupled interactions among different phases. But on the other hand, the presence of biosurfactants would also alter the balance between the capillary and buoyancy forces. Therefore, a caveat is that the lowered capillary factor could facilitate more gravity segregation due to the enhanced buoyancy effect. It necessitates follow-up experimental (core-scale) and numerical efforts (field-scale) to comprehensively investigate consequences of capillary factor control using biosurfactants on sweep efficiencies, relative permeabilities, injectivities, pore-pressure buildup, and ensuing stress changes.

#### Implications to GCS

The existence of indigenous biosurfactant-producing microorganisms, such as *Pseudomonas aeruginosa* and *B. subtilis*, can be considered one among the important parameters when prospective GCS candidate sites are selected (Maier and Soberon-Chavez, 2000). *B. subtilis*, one of the most studied strains of genus *Bacillus*, has been widely studied for potential applications in engineering practices such as microbial enhanced oil recovery and bioremediation (Banat et al., 2000; Zhang et al., 2007; Joshi et al., 2008; Vaz et al., 2012). While *Pseudomonas aeruginosa* is proven to produce a biosurfactant, rhamnolipid, the survivability of *Pseudomonas* spp. in the presence of supercritical CO_2_ remains unclear. Recently, microbial activity by halophilic and sulfate-reducing bacteria was reported in the shallow geological formations at Ketzin, Germany, a GCS candidate site of which depth, pressure and temperature are ∼700–850 m, ∼6.2 MPa and 35°C, respectively (Morozova et al., 2010). In such sites, injection of nutrients that can stimulate the indigenous microorganisms to produce biosurfactants can be utilized as one of the possible strategies prior to CO_2_ injection. Meanwhile, it is worth noting that the unexpected growth of indigenous microorganisms should also be considered as a risk factor when implementing *in situ* biosimulation; for instance, nutrient injection can stimulate the growth of sulfate reducing bacteria (SRB) that can cause H_2_S production as well as the spore-forming *Bacillus* species. In addition to the CO_2_ phase and P/T condition that were observed to influence the effectiveness of biosurfactants, other factors, such as rock mineralogy, salinity and pH of pore water, and biosurfactant type, can also affect the extent of the alteration in IFT and wettability by microbial activities. For instance, the baseline IFT and contact angle of CO_2_-brine-quartz are known to increase as the brine salinity increases (Bachu and Bennion, 2008; Jung and Wan, 2012). Although previous studies evaluated the stability of lipopeptide surfactant, surfactin under extremely saline conditions of 2.1–15.9% (Al-Wahaibi et al., 2014; Liu et al., 2015), the effect of pH and the salinity of brine on the bacterial alteration in the IFT and contact angle needs further investigations because the salinity of our mineral salt media was as low as approximately 1.6–1.7% and it represents rather low-salinity brine.

## Conclusion

This study investigated the potential of microbial biosurfactant produced at high pressure conditions as an enhancer of GCS. The extent of CO_2_-brine IFT reduction and CO_2_ wettability modification caused by *B. subtilis* was quantified for three phases of CO_2_. For all three phases of CO_2_, the CO_2_-brine IFT values decreased and the contact angles of CO_2_-brine-quartz increased with the surfactin production, but soon those were restricted by the lower limits and the upper limits, respectively, possibly due to their CMC. While the variations in IFT and contact angle differed with the phase of CO_2_, temperature and pressure, it appeared that those relative changes increased with an increase in CO_2_ density; hence liquid CO_2_ resulted in the greatest alterations in IFT, contact angle, and capillary factor. Owing to the reduced capillary factors by the produced surfactin production, the enhancement in the sweep efficiency by CO_2_ was observed via flow simulations using PNMs. The improvement was the most for liquid CO_2_ as ∼18%, and the least in supercritical CO_2_ as ∼11%. Given the existence of indigenous biosurfactant producing microorganisms in GCS sites, our study provides insights to feasibility of bio-stimulation technique to enhance CO_2_ storage capacity or injection strategy for GCS though the risks of unexpected microbial activities, such as H_2_S generation by SRB, should be further evaluated. Our study can be also extended to implementation of biosurfactant produced *ex situ* to GCS as a way to improve the CO_2_ displacement and sweep efficiency.

## Author Contributions

TP and TK designed the experiments, and TP and HJ performed the tests and interpreted the test results. GK and SK developed the PNM simulation. SY provided the microbial analysis and interpretation. TP and TK wrote the manuscript as the main authors, and SY and SK edited the manuscript.

## Conflict of Interest Statement

The authors declare that the research was conducted in the absence of any commercial or financial relationships that could be construed as a potential conflict of interest.
